# Retrospective study of peptide receptor radionuclide therapy for Japanese patients with advanced neuroendocrine tumors

**DOI:** 10.1002/jhbp.1014

**Published:** 2021-07-14

**Authors:** Noritoshi Kobayashi, Damian Wild, Felix Kaul, Takeshi Shimamura, Shoko Takano, Yuma Takeda, Naoki Okubo, Akihiro Suzuki, Motohiko Tokuhisa, Yasushi Ichikawa

**Affiliations:** ^1^ Department of Oncology Yokohama City University Graduate School of Medicine Yokohama Japan; ^2^ Division of Nuclear Medicine University Hospital Basel Basel Switzerland; ^3^ Shimamura Clinic Kawasaki Japan; ^4^ Department of Radiation Oncology Yokohama City University Graduate School of Medicine Yokohama Japan

**Keywords:** neuroendocrine tumor, peptide receptor radionuclide therapy, somatostatin receptor scintigraphy

## Abstract

**Background:**

Peptide receptor radionuclide therapy (PRRT) with radiolabeled somatostatin analogs is an innovative treatment for advanced somatostatin‐positive neuroendocrine tumors (NETs). PRRT cannot be performed in Japan because there is no approval or insurance cover so far.

**Methods:**

We relied on foreign institutions to perform PRRT for Japanese patients with NETs. We retrospectively evaluated the safety and efficacy of PRRT. The inclusion criteria were pathologically confirmed well‐differentiated NET and visible tumor uptake on pre‐therapeutic somatostatin receptor scintigraphy. ^177^Lu‐DOTA‐TOC was used as the standard treatment, and patients received three infusions every 8 weeks. Until the end of 2017, combination treatment with ^90^Y and ^177^Lu‐DOTA‐TOC was performed using the same protocol.

**Results:**

Thirty‐five patients were evaluated, and the primary lesions were pancreas, rectum, small intestine, stomach, and other locations. The partial response rate was 42.9%. Progression‐free survival (PFS) was 12.8 months and overall survival was 42.8 months. There was no significant difference in PFS between front‐line and late‐line PRRT (11.0 months vs 28.0 months; *P* = .383). Severe adverse events included lymphocytopenia (20.0%) and thrombocytopenia (5.7%). Myelodysplastic syndrome occurred in one case.

**Conclusion:**

PRRT was effective and safe for Japanese patients with advanced NETs. PRRT was equally effective as front‐line and late‐line treatment.

## Graphical abstract

**Highlight**

Peptide receptor radionuclide therapy with radiolabeled somatostatin analogs is an innovative treatment for advanced somatostatin‐positive neuroendocrine tumors. This retrospective study by Kobayashi and colleagues, which constitutes the first report of peptide receptor radionuclide therapy for Japanese patients with advanced neuroendocrine tumors, demonstrated good efficacy and safety in the Japanese patient cohort.

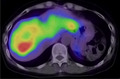

## 1 INTRODUCTION

Neuroendocrine tumors (NETs) are a heterogeneous group of neoplasms with predominantly neuroendocrine differentiation that can occur almost anywhere in the human body.^1^ Most of these neoplasms express somatostatin receptor subtype 2 (SSTR2), which are important targets for therapy and diagnosis.^2^, ^3^ Somatostatin analogs, such as octreotide LAR and lanreotide, are effective treatment options for controlling tumor progression and hormone function in patients with NETs.^4^, ^5^ Radiolabeled somatostatin analogs are important imaging and therapeutic options. Somatostatin‐based peptide receptor radionuclide therapy (PRRT) was introduced in the 1990s in Europe.^6^, ^7^ Since then, it has developed into a valuable therapeutic tool for patients with NETs. The introduction of tyrosine into the third position of the octreotide sequence increased the hydrophilicity and receptor affinity of the peptide, and conjugation with the beta emitter ^90^Y allowed irradiation of the tumor.^8^ ^90^Y‐DOTA‐TOC is administered intravenously, binds to somatostatin receptors on the target cell, and causes cytotoxic effects via beta irradiation. The current practice is the use of a single radioisotope, mainly ^177^Lu. ^177^Lu has a short‐range, low‐energy beta emission that allows the concentration of most of its dose in the tumor lesions and not in the surrounding tissue.^9^ PRRT was evaluated in many retrospective and small prospective clinical trials, but only recently, a randomized phase III study was performed with ^177^Lu‐DOTATATE in patients with midgut NETs.^10^ ^177^Lu‐DOTATATE was approved by the FDA for the treatment of somatostatin receptor‐positive NETs in 2018. However, somatostatin‐based PRRT has not yet been approved in Japan. We have been performing PRRT in collaboration with the University Hospital of Basel since 2011. Here, we retrospectively report the safety and efficacy of PRRT with ^90^Y‐/^177^Lu‐DOTA‐TOC in our Japanese patient cohort.

## 2 MATERIAL AND METHODS

### 2.1 Study design

This is a retrospective and longitudinal observational study in which the impact on the morphological response, progression‐free survival (PFS), overall survival (OS), toxicity, and prognostic factors were evaluated in patients with advanced somatostatin receptor‐expressing NETs. The main objective of this retrospective study was to evaluate PFS as the primary endpoint. The relevant information of this patient cohort was collected by obtaining clinical information, including treatment response and disease status. The data were anonymized for further analyses. This clinical trial was approved by the Institutional Research Board of the Yokohama City University Hospital (IRB B180100019).

### 2.2 Patients

Eligible patients were more than 20 years old and were consecutively enrolled with histologically confirmed advanced well‐differentiated NETs with SSTR2 expression. Somatostatin receptor scintigraphy (SRS) with ^111^In‐Pentetoreotide or ^68 Ga^‐DOTA‐TOC‐PET/CT was performed, and baseline tumor uptake on SRS in tumor cells had to be at least as high as in normal liver tissue (Krenning scale >2) as a requirement for PRRT inclusion.^11^ Confirmation of SSTR2 expression in the tumor tissue of the first four patients was performed using immunohistochemistry because there was no national insurance cover for SRS in Japan before 2015. Histological evaluation of SSTR2 was performed according to the Volante score (score >2).^12^ Different treatments were conducted before PRRT, including surgical resection, somatostatin analogs (octreotide and lanreotide), molecular targeted therapies (everolimus and sunitinib), cytotoxic chemotherapy (streptozocin, capecitabine, fluorouracil, and temozolomide), target therapies (trans‐arterial chemoembolization [TACE] and radiofrequency ablation [RFA]), and extra‐beam radiation (conventional irradiation and proton beam). However, we excluded patients with concurrent antitumor treatments. Treatment with long‐acting somatostatin analogs was stopped 6 weeks before PRRT, and molecular targeted therapy and cytotoxic chemotherapy were stopped 4 weeks before PRRT. We excluded pregnancy, breast‐feeding, urinary incontinence, pre‐existing hematologic toxicities grades 3 to 4, and severe concomitant illness, including severe psychiatric disorders. We excluded patients who had already received PRRT.

The inclusion criteria were Eastern Cooperative Oncology Group (ECOG) performance status <2, and patients were able to travel long distances to Switzerland. Adequate bone marrow, renal and hepatic functions were required (white blood cell count >3000/L, hemoglobin count >8.0 mg/L, platelet count >90 000/L, creatinine <1.5, bilirubin level <3 × upper limit). Patients with hormone‐active NETs were asked to stop long‐acting somatostatin analogs 6 weeks before PRRT, which were allowed to switch to short‐acting somatostatin analogs for up to 24 h before PRRT. Restart with somatostatin analogs was allowed 2 days after PRRT.

### 2.3 Treatment

DOTA‐TOC was synthesized using a five‐step synthetic procedure according to good laboratory practice at the University Hospital of Basel.^13^, ^14^ Alternate treatment with ^90^Y‐DOTA‐TOC and ^177^Lu‐DOTA‐TOC was performed until December 2017. Subsequently, only ^177^Lu‐DOTA‐TOC mono treatments were performed. The treatment activity was 3.7 GBq/m^2^ body surface of ^90^Y plus 0.111 GBq of the gamma emitter ^111^Indium, which was used for post‐therapeutic imaging or with 7.4GBq of ^177^Lu. The radioisotopes were incubated with lyophilized DOTA‐TOC kits for 30 min at 95℃. Quality control was performed using solid‐phase extraction and high‐performance liquid chromatography, with a minimum required radiochemical purity of 95%. An infusion of 1000 mL physiological NaCl solution containing 20.7 mg/mL of arginine and 20.0 mg/mL of lysine was started 30 min before and continued for 4 h after ^90^Y‐/^177^Lu‐DOTA‐TOC injection to inhibit tubular reabsorption of the radiopeptide. Patients were hospitalized two to three nights for each cycle, following the Swiss requirements for legal radiation protection. Normally, three treatment cycles were performed at an interval of at least 6 weeks. The treatment was not covered by Japanese health insurance support. Therefore, the patient paid about 11 000 EURO per one treatment.

### 2.4 Follow‐up

The patients returned to Japan as soon as possible after discharge from the University Hospital of Basel. Domestic hospital visits were scheduled every 2 weeks in order to check their physical condition, and to get a blood test, including blood count, liver values, and creatinine. Treatment cycles were postponed for up to 4 weeks if the physical condition and laboratory results of the patient did not meet the minimal requirements for the continuation of the next treatment cycle. Furthermore, PRRTs were stopped if blood values did not improve even after the prolongation of the treatment interval or if the patient's condition did not allow the continuation of additional treatment cycles. A CT scan was performed 8‐10 weeks after the last treatment cycle. We defined the morphological findings as complete response (CR), partial response (PR), stable disease (SD), and progressive disease (PD) according to RECIST criteria version 1.1.^15^ We recommend alternative treatment options for PD. In the case of SD, PR, and CR, a wait‐and‐watch strategy was recommended. We started or restarted treatment with long‐acting somatostatin analogs as maintenance treatment in all patients with functional and some with non‐functional NETs.

### 2.5 Outcomes and statistical analysis

The main objective of this retrospective study was to evaluate PFS as the primary endpoint. PFS was defined from the date of the first PRRT to disease progression or the time of another treatment. We also analyzed response rate (RR), disease control rate (DCR), and OS. RR was defined as the CR plus PR, and DCR was defined as the CR plus PR plus the rate of patients with SD for the follow‐up period according to RECIST criteria version 1.1. OS was defined as the time from the start of the first PRRT treatment until death. Patients who were alive at the time of the final analysis or who had been lost to follow‐up were censored at their last known alive data.

Toxicity was evaluated according to the NCI CTCAE criteria version 4 ^16^. Acute and subacute (up to 72 h and 2 months after each radiolabeled DOTATOC administration, respectively) side‐effects were recorded. Blood counts, liver and kidney biochemistry were performed just prior to the radiolabeled DOTATOC treatment and 2, 4, and 6 weeks after each cycle, as well as 3 months after the induction course and as per routine clinical follow‐up thereafter. Patients who stopped therapy before the third cycle for any reason other than PD were also considered evaluable for treatment activity and safety analysis.

Statistical analysis was performed using the IBM SPSS version. A descriptive analysis was carried out to describe the continuous variables as means, medians, and standard deviation, while categorical variables were described as proportions, including 95% confidence intervals (CI). Comparisons between subgroups were performed with the Kruskal‐Wallis test for continuous variables and with the χ^2^ test for non‐continuous variables. PFS and OS were estimated using the Kaplan‐Meier method.

## 3 RESULTS

### 3.1 Patient's characteristics

PRRT was performed on 38 patients between 2011 and 2019. We excluded three patients from this study (two patients received PRRT before this protocol, and one patient could not receive the full treatment activity because of worse performance status and severe bone and liver metastases). A total of 35 patients were included in this study. The patient characteristics are described in Table 1. The median age at the first PRRT was 57 years (age range: 26‐70 years); 18 patients were male, and 17 patients were female. The performance status was 0 or 1. The average period from diagnosis to the first PRRT was 31.8 months (range: 4.8‐93.1 months). The primary lesions were in the pancreas (n = 20, 57.1%), rectum (n = 6, 17.1%), small intestine (n = 3, 8.6%), stomach (n = 1, 2.9%), lung (n = 1, 2.9%), and thymus (n = 1, 2.9%). Three patients (8.6%) were unknown. Thirty‐three patients had tumor progression at baseline, and two patients showed SD at baseline. Most of the patients had liver metastases, followed by metastases in the lymph nodes and bones. Before PRRT patients received surgical resection (n = 20, 57.1%), treatment with somatostatin analogs (n = 32, 91.4%), molecular targeted therapy (n = 13, 37.1%), cytotoxic chemotherapy (n = 14, 40%), and extra‐beam radiation therapy (n = 4, 11.4%). The median number of treatments before PRRT was two (range: 0‐13). The median follow‐up period was 29.9 months (range: 4.8‐93.1 months). Four patients had a history of a hormone‐active NET (gastrinoma, n = 2; insulinoma, n = 1; VIPoma, n = 1), eight patients had diabetes, and six patients had secondary cancer in their past medical history (lung cancer, n = 2; thyroid cancer, n = 2; breast cancer, n = 1; basal cell carcinoma, n = 1) and MEN type 1 in one case. All patients with a secondary tumor remained in complete remission for the duration of this study. The median Ki67 index was 6% (range: 0.7%‐30%). According to the 2019 WHO classification, there were three grade 1, 29 grade 2, and three grade 3 cases. In all patients, reassessment of tissue samples at our institution confirmed the well‐differentiated nature of the NETs. The three patients with histologically confirmed NET G3 had a Ki67 index of 20%, 21%, and 30%.

**TABLE 1:** Patient characteristics

	All cases (n = 35)	^90^Y ‐/^177^Lu –DOTA‐TOC combination (n = 16)	^177^Lu‐DOTA‐TOC monotherapy (n = 19)
Gender, n (%)			
Male	18 (51.4%)	6 (37.5%)	12 (63.2%)
Female	17 (48.6%)	10 (62.5%)	7 (36.8%)
Age (years), mean (SD)	57 (26‐70)	54.5 (26‐68)	62 (34‐70)
Primary tumor site, n (%)			
Pancreas	20 (57.1%)	10 (62.5%)	10 (52.6%)
Stomach	1 (02.9%)	00 (00.0%)	01 (05.3%)
Small intestine	3 (08.6%)	02 (05.7%)	01 (05.3%)
Rectum	6 (17.1%)	03 (08.6%)	03 (15.8%)
Others	5 (14.3%)	01 (02.9%)	04 (21.1%)
Site of metastasis, n (%)			
Liver	33 (94.2%)	15 (93.8%)	18 (94.7%)
Lymph nodes	20 (57.1%)	09 (56.3%)	11 (57.9%)
Bone	11 (31.4%)	05 (31.3%)	06 (31.6%)
Lungs	2 (5.7%)	01 (06.3%)	01 (05.3%)
Pathological Classification			
NET Grade 1	3 (8.6%)	1 (6.3%)	2 (10.5%)
NET Grade 2	29 (82.9%)	14 (87.5%)	15 (78.9%)
NET Grade 3	3 (8.6%)	1 (6.3%)	2 (10.5%)
SRS, Krenning scale, n (%)^a^	0		
Grade 2	4 (12.9%)	00 (00.0%)	04 (22.2%)
Grade 3	06 (19.4%)	02 (07.7%)	04 (22.2%)
Grade 4	21 (67.7%)	11 (84.6%)	10 (55.5%)
Progression at baseline	33 (94.3%)	16 (100%)	17 (89.5%)
Functional NET	4 (11.4%)	1 (6.3%)	3 (15.8%)
Period from diagnosis to PRRT	31.8 months (4.8‐93.1)	42.8 months (9.9‐93.1)	21.5 months (4.8‐60.3)
Previous treatment number (included surgical resection )	2 (0‐13)	2 (0‐13)	2 (0‐8)
Previous surgical resection	20/35 (57.1%)	11/16(68.8%)	9/19(47.4%)
Previous treatment Somatostatin analog	32/35 (91.4%)	13/16 (81.2%)	19/19 (100%)
Previous treatment Molecular targeted therapy	13/35 (37.1%)	4/16 (25%)	9/19 (47.4%)
Previous treatment Chemotherapy	14/35 (40%)	8/16 (50%)	6/19 (32.6%)

Abbreviations: Chemotherapy (eg Streptozocine, Temozolomide); Molecular targeted therapy: (eg Everolimus, Snitinib); NET, Neuroendocrine tumor; PRRT, Riptide receptor radionuclide therapy; Somatostatin analog (eg Octreotide, Lanreotide); SRS, Somatostatin receptor Scintigraphy.

^a^ Four cases did not perform SRS before PRRT.

The modified maximum Krenning scale of pre‐treatment SRS was 0 (n = 0), 1 (n = 0), 2 (n = 4), 3 (n = 6), and 4 (n = 21).

### 3.2 Treatment

Twenty‐eight patients received PRRT three times as initially planned (Tables 2,3). Seven patients could not complete all three cycles of treatment, five patients received PRRT twice, and two patients received PRRT once. The reasons for not completing treatment were as follows: tumor progression (five patients), thrombocytopenia (one patient), and uncontrollable hormonal symptoms (one patient). In the combination group, patients received a median dose of 4.625 (range: 3.7‐5.55) GBq of ^90^Y‐DOTA‐TOC or a median dose of 5.55 (range: 5.55‐7.4) GBq ^177^Lu‐DOTA‐TOC per single injection according to their physical conditions. In the monotherapy group, the median dose of ^177^Lu was 5.55 (range: 5.55‐7.4) GBq per single injection according to their physical conditions.

**TABLE 2:** Treatment dose (Combination Group)

Dose of ^90^Y‐/^177^Lu‐DOTA‐TOC combination therapy		Number of Patients
^90^Y‐DOTA‐TOC	Median 4.625 (3.7‐5.55) GBq	
^177^Lu‐DOTA‐TOC	Median 5.55 (5.55‐7.4) GBq	

Number of administrations, n (%)	
3	14 (87.5%)
2	1 (6.3%)
1	1 (6.3%)
0	0 (0.0%)

Abbreviation: GBq; Gigabecquerel.

**TABLE 3:** Treatment dose (Monotherapy Group)

Dose of ^177^Lu‐DOTA‐TOC monotherapy		Number of Patients
^177^Lu‐DOTA‐TOC	Median 5.55 (5.55‐7.4) GBq	

Number of administrations, n (%)	
3	14 (73.7%)
2	4 (21.0%)
1	1 (5.3%)
0	0 (0.0%)

Abbreviation: GBq; Gigabecquerel.

### 3.3 Efficacy

**FIGURE 1:**
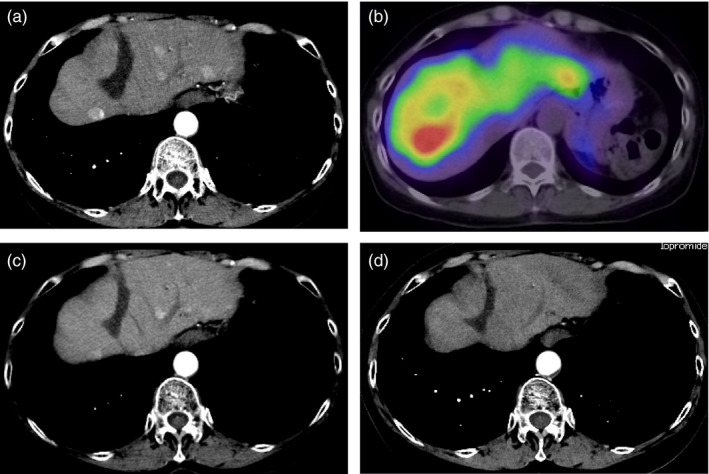
Representative case treated with PRRT. Pancreatic NET with multiple liver metastasis post distal pancreatectomy and multiple liver resection state (WHO 2017 grade 2). (A) Enhanced CT findings revealed multiple hyper vascular lesions in liver before the PRRT. (B) SPECT‐CT imaging before the PRRT. Moderate to intensity uptake revealed multiple liver metastatic lesions. (C) Enhanced CT findings revealed multiple hyper vascular lesions in liver were slightly decrease in size ten weeks after the final PRRT. (D) Enhanced CT findings revealed multiple hyper vascular lesions in liver almost disappeared 28 mo after the final PRRT

All patients were assessed for imaging responses. The RR was 42.9% in all patients (CR: n = 0, 0%; PR: n = 15, 42.9%). Another 20.0% of patients (n = 7) showed SD. The DCR was 62.9%. The RR was 45.4% and the DCR was 48.5% in baseline progressive patients (n = 33). In patients with pancreatic NETs the RR was 45% (CR: n = 0; PR: n = 9: SD: n = 4; PD: n = 7), in patients with gastrointestinal NETs the RR was 60% (CR: n = 0; PR: n = 6; SD: n = 0; PD: n = 4), and in the remaining patient, the RR was 0% (CR: n = 0; PR: n = 0; SD: n = 3; PD: n = 2; Tables 4,5). The RR was 56.2% in the ^90^Y‐/ ^177^Lu‐DOTA‐TOC group (CR: n = 0; PR: n = 9; SD: n = 3; PD: n = 4) and 31.6% in the ^177^Lu‐DOTA‐TOC monotherapy group (CR: n = 0; PR: n = 6; SD: n = 4; PD: n = 9). We described a representative case (Figure 1A, B, C, D). A Japanese female aged in her 60s received distal pancreatectomy with segmental liver resection. Pathological findings revealed well‐differentiated neuroendocrine tumor (WHO2017: grade 2). After 6 months, multiple liver metastatic lesions appeared, and she received somatostatin analogue for one year. However, multiple liver lesions had increased in size. Somatostatin receptor scintigraphy revealed moderate to intense uptake in liver lesions (Krenning scale: score 3 and 4). She received three PRRTs with ^177^Lu‐DOTA‐TOC (5.55GBq) and maintenance somatostatin analogue treatment. CT findings revealed multiple hyper vascular lesions in liver were slightly decreased in size, and partial response was achieved 12 months after the final PRRT. Multiple hyper vascular lesions in liver almost disappeared 28 months after the final PRRT.

**TABLE 4:** Response rates in treatment

	All cases (n = 35)	^90^Y‐/^177^Lu‐DOTA‐TOC combination (n = 16)	^177^Lu‐DOTA‐TOC monotherapy (n = 19)
Complete Response (n, %)	0	0	0 (%)
Partial Response (n,%)	15 (42.9%)	9 (56.2%)	6 (31.6%)
Stable Disease (n, %)	7 (20.0%)	3 (18.8%)	4 (21.0%)
Progressive Disease (n, %)	13 (37.1%)	4 (25.0%)	9 (47.4%)

**TABLE 5:** Response rates in primary lesion

	All cases (n = 35)	Pancreas (n = 20)	GI‐tract (n = 10)	Others (n = 5)
Complete Response (n, %)	0 (0%)	0 (0%)	0 (%)	0 (0%)
Partial Response (n, %)	15 (42.9%)	9 (45%)	6 (60%)	0 (0%)
Stable Disease (n, %)	7 (20.0%)	4 (20%)	0 (0%)	3 (60%)
Progressive Disease (n, %)	13 (37.1%)	7 (35%)	4 (40%)	2 (40%)

**FIGURE 2:**
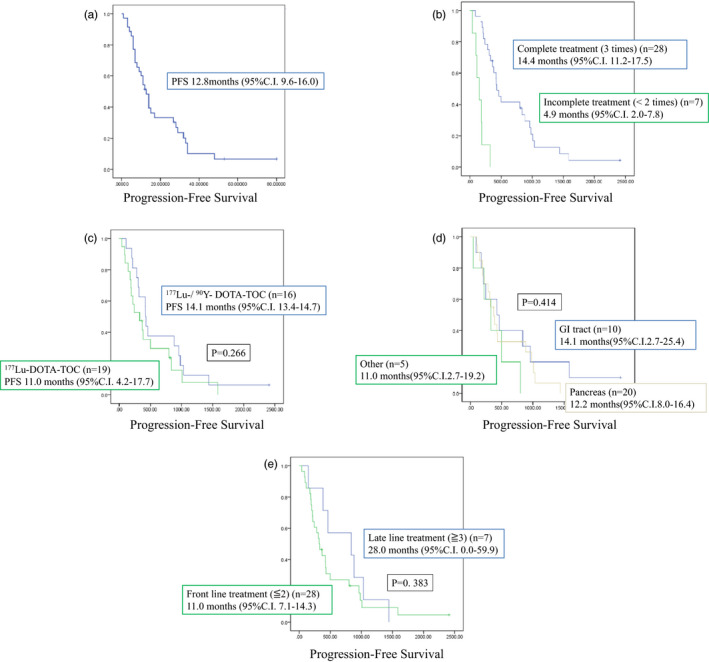
Progression free survival. (A) Progression free survival of all patients. (B) Comparison of complete treatment and incomplete treatment. (C) Comparison of the ^90^Y‐/ ^177^Lu‐DOTA‐TOC Combination treatment and ^177^Lu‐DOTA‐TOC monotherapy. (D) Comparison of primary lesions. (E) Comparison of front‐line treatment and late line treatment

The median follow‐up period from the start of treatment was 29.9 months (range: 4.8‐93.1 months). The median follow‐up period after the last therapy was 25.5 months (range: 2.5‐88.4 months). The median PFS was 12.8 months (95%CI, 9.0‐16.5 months) in all cases (Figure 2A), 14.4 months (95%CI, 11.2‐17.5 months) in complete treatment cases (Figure 2B), and 12.8 months (95%CI, 8.2‐17.3 months) in baseline progressive cases. The median PFS 14.1 months in the ^90^Y‐/^177^Lu‐DOTA‐TOC group (95%CI, 13.4‐14.7 months) and 11.0 months in the ^177^Lu‐DOTA‐TOC monotherapy group (95%CI, 4.2‐17.7 months; Figure 2C). There was no significant difference between the two groups (*P* =.266). PFS values were not significantly different in patients with pancreatic NETs (12.2 months,95% CI, 8.0‐16.4 months), in patients with gastrointestinal NETs (14.1 months, 95% CI, 2.7‐25.4 months), and in the remaining patients (11.0 months, 95% CI, 2.7‐19.2 months; *P* =.414; Figure 2D).

We compared PFS in patients who received PRRT as front‐line treatment (first‐line or second‐line treatment) and late‐line treatment (third‐line or fourth‐line). PFS was 11.0 months (95% CI, 7.1‐14.3 months) and 28.0 months (95% CI, 0‐59.9 months) in the front‐line treatment and the late‐line treatment groups, respectively (Figure 2E). There was no significant difference between the two groups (*P* =.383). PFS was also not significantly different (*P* =.159) between patients who received PRRT after molecular targeted therapy (7.2 months, 95% CI, 0‐15.0 months) and those patients who received PRRT before starting with the molecular targeted therapy (14.1 months, 95% CI, 10.9‐17.3 months; Figure S1). PFS was not significantly different (*P* =.375) between PRRT followed by chemotherapy (14.1 months, 95% CI, 8.6‐19.5 months) and PRRT before chemotherapy (11.0 months, 95% CI, 6.8‐15.2 months; Figure S2). Nine patients received somatostatin analogs as maintenance treatment after PRRT. PFS was not significantly different between patients who received maintenance treatment and those who did not (26.7 months; 95% CI, 11.6‐41.8 months vs. 10.2 months; 95% CI, 6.6‐13.9 months; Figure S3). There was a tendency that maintenance treatment with somatostatin analogs is beneficial. However, the results were not significantly better in this study with a limited number of patients (*P* =.071).

**FIGURE 3:**
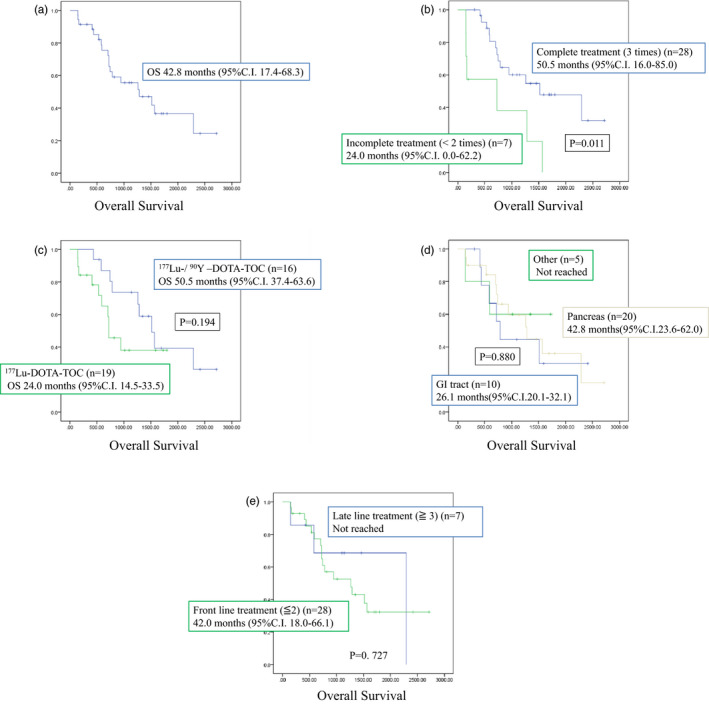
Overall survival. (A) Overall survival of all patients. (B) Comparison of complete treatment and incomplete treatment. (C) Comparison of the ^90^Y‐/^177^Lu‐DOTA‐TOC Combination treatment and ^177^Lu‐DOTA‐TOC monotherapy. (D) Comparison of primary lesions. (E) Comparison of front‐line treatment and late line treatment

The median OS was 42.8 months (95% CI, 17.4‐68.3 months) in all patients (Figure 3A), 50.8 months (95% CI, 16.0‐85.0 months) in complete treatment cases and 24.0 months (95% CI, 0.0‐62.2 months) in incomplete treatment cases (*P* =.011; Figure 3B). The median OS was 42.0 months (95% CI, 17.1‐66.9 months) in baseline progressive cases. The median OS was 50.5 months (95% CI, 37.4‐63.6 months) in the ^90^Y‐/^177^Lu‐DOTA‐TOC treated group and 24.0 months in the ^177^Lu‐DOTA‐TOC monotherapy group (95%CI, 14.5‐33.5 months; Figure 3C). There was no significant difference between the two groups (*P* =.194).

OS was 42.8 months (95%CI, 23.6‐62 months) in patients with pancreatic NETs and 26.1 months (95%CI, 20.1‐32.1 months) in patients with gastrointestinal NETs (Figure 3D). There was no significant difference between the two groups (*P* =.880).

OS was 42.0 months (95% CI, 18.0‐66.1 months) in patients who received PRRT as front‐line treatment and 76.3 months (95% CI, 28.0‐84.7 months) in patients who received PRRT as late‐line treatment (Figure 3E). There was no significant difference between the two groups (*P* =.727).

OS was not significantly different, whether PRRT followed molecular targeted therapy or not (24.0 months; 95% CI, 17.5‐30.5 months vs. 52.2 months; 95%CI, 33.8‐70.5 months; *P* =.108; Figure S4), and OS was not significantly different between PRRT, whether followed by cytotoxic chemotherapy or not (52.2 months; 95%CI, 2.3‐102.0 months vs. 42.0 months; 95%CI, 21.2‐63.2 months; *P* =.998; Figure S5).

OS was not significantly different in patients who received maintenance treatment with somatostatin analogs and not after PRRT (42.8 months; 95%CI, 14.8‐70.9 vs. 42.8 months; 95%CI, 17.4‐70.9; *P* =.384; Figure S6).

### 3.4 Adverse events

Adverse events (AEs) were evaluated using CTCAE version 4.0. Tables 6,7 show acute and subacute hematological and non‐hematological toxicities. In all grades, lymphocytopenia (65.7%), anemia (48.6%) and thrombocytopenia (48.6%) were the most frequently observed hematological toxicities. The most frequently observed severe toxicity was lymphocytopenia (20%), however, the rate of other hematological toxicities was under 10%. In all grade non‐hematological toxicities, liver dysfunction (40%), nausea (45.7%), general fatigue (31.4%), and appetite loss (37.1%) were the most observed toxicities. However, severe non‐hematological toxicities were rare. There were no cases of treatment‐related death. We compared the rates of AEs in the ^90^Y‐/^177^Lu‐DOTA‐TOC and ^177^Lu‐DOTA‐TOC monotherapy groups. There was no significant difference of hematological toxicity between the two groups. General fatigue was the only non‐hematological adverse effect that was significantly more frequently reported in patients who received ^90^Y‐/^177^Lu‐DOTA‐TOC than in patients who received ^177^Lu‐DOTA‐TOC monotherapy (*P* =.035).

**TABLE 6:** Hematological toxicities

	Grade 3,4 n (%) Total Case ^177^Lu‐/^90^Y‐ DOTA‐TOC ^177^Lu‐DOTA‐TOC	All Grade n (%) Total Case ^177^Lu‐/^90^Y‐ DOTA‐TOC ^177^Lu‐DOTA‐TOC	*P* value
Anemia	1/35 (2.9) 1/16 (6.3) 0/19 (0)	17/35 (48.6) 9/16 (56.3) 8/19 (42.1)	.404
Leucopenia	2/35 (5.7) 2/16 (12.5) 0/19 (0)	14/35 (40) 9/16 (56.3) 5/19 (26.3)	.072
Neutropenia	0/35 (0) 0/16 (0) 0/19 (0)	13/35 (37.1) 8/16 (50) 5/19 (26.3)	.149
Lymphocytopenia	7/35 (20) 4/16 (25) 3/19 (15.8)	23/35 (65.7) 11/16 (68.8) 12/19 (63.2)	.728
Thrombocytopenia	2/35 (5.7) 2/16 (12.5) 0/19	17/35 (48.6) 10/16 (62.5) 7/19 (36.8)	.130
Febrile Neutropenia	0/35 (0)	0/35 (0)	

Note

Late hematological Toxicity. n = 1 (2.9%).

Abbreviation: MDS, Myelodysplastic syndrome.

**TABLE 7:** Non‐hematological toxicities

	Grade 3,4 n (%) Total ^177^Lu‐/^90^Y‐ DOTA‐TOC ^177^Lu‐DOTA‐TOC	All Grade n (%) Total ^177^Lu‐/^90^Y‐ DOTA‐TOC ^177^Lu‐DOTA‐TOC	*P*‐value
Liver dysfunction (AST)	1/35 (2.9) 1/16 (6.3) 0/19 (0)	14/35 (40) 7/16 (43.8) 7/19 (36.8)	.678
Liver dysfunction (ALT)	1/35 (2.9) 0/16 (0) 1/19 (5.3)	10/35 (28.6) 5/16 (31.3) 5/19 (26.3)	.748
Renal dysfunction	0/35 (0) 0/16 (0) 0/19 (0)	6/35 (17.1) 1/16 (6.3) 5/19 (26.3)	.117
Fever	1/35 (2.9) 0/16 (0) 1/19 (5.3)	1/35 (2.9) 0/16 1/19 (5.3)	.352
Nausea	1/35 (2.9) 0/16 (0) 1/19 (5.3)	16/35 (45.7) 9/16 (56.3) 7/19 (36.8)	.251
Vomiting	1/35 (2.9) 0/16 (0) 1/19 (5.3)	7/35 (20) 2/16 (12.5) 5/19 (26.3)	.309
Diarrhea	1/35 (2.9) 0/16 (0) 1/19 (5.3)	7/35 (20) 3/16 (18.8) 4/19 (21.1)	.865
Constipation	0/35 (0) 0/16 (0) 0/19 (0)	1/35 (2.9) 0/16 (0) 1/19 (5.3)	.352
General fatigue	0/35 (0) 0/16 (0) 0/19 (0)	11/35 (31.4) 8/16 (50) 3/19 (15.8)	.035
Appetite loss	1/35 (2.9) 0/16 (0) 1/19 (5.3)	13/35 (37.1) 7/16 (43.8) 6/19 (31.6)	.458
Alopecia	0/35 (0) 0/16 (0) 0/19 (0)	1/35 (2.9) 0/16 (0) 1/19 (5.3)	.352
Neuropathy	0/35 (0) 0/16 (0) 0/19 (0)	1/35 (2.9) 1/16 (6.3) 0/19 (0)	.269

One patient developed myeloid dysplastic syndrome (MDS). This patient received ^90^Y‐/^177^Lu‐DOTA‐TOC. No other severe late phase AEs were observed.

## 4 DISCUSSION

In this study, we report for the first time the retrospective outcome data of PRRT in Japanese patients with advanced NETs. Importantly, the objective RR was the same or slightly higher in our study than in previous studies ^9^, ^10^, ^17^, ^18^, ^19^, ^20^, ^21^. The RR of the data in previous reliable studies was 18%–42%. One possible reason for the high objective RR might be the high radiotracer uptake in tumor lesions of most patients in pretreatment somatostatin receptor scans. There is evidence for a correlation between high tumor uptake and good treatment response ^13^. This correlation allows prediction of treatment response which is an important advantage of PRRT.

On the other hand, PFS tended to be shorter than reported in previous studies ^9^, ^10^, ^17^, ^18^, ^19^, ^20^, ^21^. PFS of the data in previous reliable studies was 28.4‐33 months. One reason for this finding is most likely the lower number of treatment cycles (three cycles) than in other studies. Consequently, the duration of the treatment is not only shorter, but also the total treatment activity is lower compared to other studies which used a standard protocol of four cycles ^22^. The long and exhausting journey from Japan to Basel and back was the main reason for performing three instead of four treatment cycles. Another reason for this finding is the early termination of the treatment in seven patients (20%) who could not receive three treatments due to their physical condition. In this study, the PFS and OS was longer in complete treatment cases than in incomplete treatment cases. At least three cycles of treatments were necessary for good PFS and long OS. However, it was a very difficult problem to predict before PRRT if the patients with high liver tumor burden could complete at least three cycles of treatment. Of course, our data were retrospective and compassed use data, so the standard protocol of four cycles should be performed as a standard treatment strategy. On the other hand, seven patients (46.7%) who achieved disease control by PRRT received re‐PRRT. Re‐PRRT was also effective in these patients (DCR 85.7%; detailed data are not shown).

OS was similar to previously reported studies, despite the advanced stage ^9^, ^10^, ^17^, ^18^, ^19^, ^20^, ^21^. OS in previous reliable studies was 30‐63 months. Most patients in our study received PRRT at a late stage (median of 31.8 months after diagnosis) because the Japanese health insurance did not cover the costs for PRRT. Furthermore, seven patients showed disease progression and aggravation of the performance status under the interval of three times PRRT. However, in these very advanced stage patients, PRRT showed a good response with good PFS and long OS. In these patients, PFS and OS were not significantly different from those who received PRRT at an earlier stage. The European Neuroendocrine Tumor Society (ENETS) recommends PRRT as a second line treatment option after failure of a treatment with somatostatin analogs only in midgut NETs. However, in the future, in certain situations, PRRT may well be considered earlier in the treatment pathway ^23^, ^24^. We also compared molecular targeted therapy and chemotherapy before and after PRRT. The sequence of PRRT (before or after molecular‐targeted therapy or chemotherapy) did not alter treatment outcomes.

In this cohort, the most frequently observed severe toxicity was lymphocytopenia, however, the rate of other severe hematological toxicities was under 10%. Liver dysfunction, nausea, general fatigue, and appetite loss were the most common observed non‐hematological toxicities, but most of the toxicities were mild and reversible. In the larger group study involving 510 patients, severe hematological toxicities occurred in 9.5%.^9^ In NETTER‐1 trial, treatment severe adverse events were reported at 9%.^10^ In the data of previous studies, the profile of toxicities, severity, and frequency resembled those of the present study of Japanese patient's data.

The safety of PRRT in patients with high tumor burden in the liver presents concerns for the potential for radiation hepatitis. However, severe liver toxicities were very rare and did not appear to correlate with baseline tumor burden in reported phase III study.^25^ However, liver tumor burden was classified into only three group (<25%, 25%–50%, >50%). Safety findings in patients with extreme tumor burden (eg,>90%) was not clear in the previous study. In our study, severe liver dysfunction occurred in only one patient in the small intestinal NET with low (<25%) liver tumor burden.

In this study, one pancreatic NET patient with multiple liver metastases developed MDS. MDS occurred 11 months after the first treatment, she did not have bone metastatic lesions and had not received chemotherapy previously. In the previous study, acute leukemia occurred in 0.7% after a median follow‐up of 55 months after the first treatment and MDS occurred in 1.5% after a median follow‐up of 28 months after the first therapy ^21^. Brieau et al reported the highest therapy‐related myeloid neoplasm incidence of up to 20% after Lu‐177 DOTATATE salvage treatment, which was evidently linked to prior exposure to alkylating agents, the number of prior therapies, and the metastatic burden in the bone.^26^

In this study, the primary tumor was mainly located in the pancreas. OS and PFS of pancreatic NETs were not significantly different from gastrointestinal NETs (PFS: 12.2 months vs 14.1 months, *P* =.414 and OS: 42.8 months vs 26.1 months, *P* =.880). In Japan, the most common gastrointestinal NET is the rectum. In addition, midgut NETs are very rare.^27^ In our study, six patients had rectal NET, and only three had a small intestinal NET. Unresectable and metastasized colorectal NETs generally have a worse prognosis than midgut NETs.^28^ In our study, the objective RR was high (66.7%), but PFS (14.1 months 95%CI 3.5‐24.5 months) and OS (24.0 months 95%CI 4.1‐32.2 months) was relatively short in rectal NETs (detail data was not shown). This epidemiological difference in patients with gastrointestinal NETs between Japan and Western countries impacts the PRRT outcome of our study.

Recently, it has been shown that maintenance treatment with somatostatin analogs after PRRT is more efficient with a longer PFS and OS than PRRT without maintenance therapy.^29^ In our study, there was a tendency for maintenance treatment with somatostatin analogs to be beneficial after PRRT. Probably due to the relatively low number of patients, the difference was not statistically significant. As in other studies, PRRT was well tolerated by our Japanese patient collective. This is not surprising, as the total radiation dose was lower than in previously reported studies. Severe AEs were relatively rare, however MDS occurred in one case. Chronic hematological toxicities, including secondary hematological malignancy, might be more frequent for a long observational duration; therefore, careful follow‐up after PRRT is required.

This study has some limitations. First, the retrospective design with a relatively small number of patients. Second, two different treatment protocols (^90^Y‐/^177^Lu‐DOTA‐TOC and only ^177^Lu‐DOTA‐TOC) were used during the observation period. Finally, the treatment activity differed between patients. The treatment activity was based on each patient's general behavior, as well as hematological and renal function.

This study summarizes, for the first time, the PRRT outcome data of Japanese patients with advanced NETs. Despite the lower number of treatment cycles compared to other studies, PRRT showed a high objective response rate, a good PFS and long OS in our Japanese patient cohort. PRRT was safe in our patients even after a long and exhausting journey. For the benefit and comfort of our patients, PRRT should become available in Japan.

## AUTHOR CONTRIBUTIONS

NK drafting of the article. FK, YT, NO, AS, MT, YH, YI: conception and design, final approval of critical revision for important intellectual content. DW: final approval of the article. All authors have read and approved the manuscript.

## Supporting information

Fig S1‐S6

Supplementary Material
