# Supplementary material for: Retrospective study of peptide receptor radionuclide therapy for Japanese patients with advanced neuroendocrine tumors
Source: J Hepatobiliary Pancreat Sci. 2021 Jul 14;28(9):727–39. doi: 10.1002/jhbp.1014 (PMC9292713; doi:10.1002/jhbp.1014)
Supplement: Supplementary file 1 — Fig S1‐S6 [file JHBP-28-727-s002.pptx]

## Slide 1
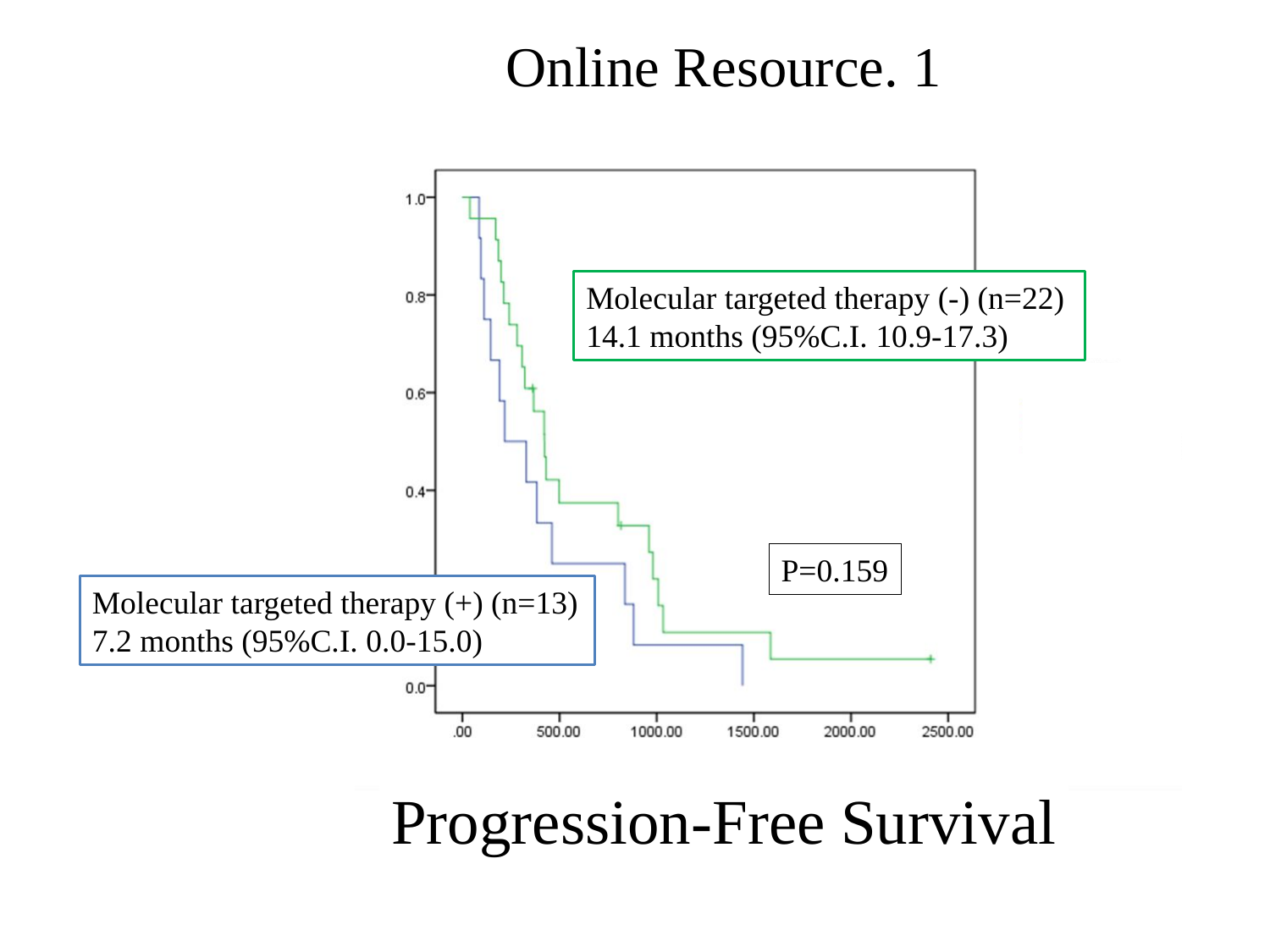

Online Resource. 1
Molecular targeted therapy (-) (n=22)
14.1 months (95%C.I. 10.9-17.3)
P=0.159
Molecular targeted therapy (+) (n=13)
7.2 months (95%C.I. 0.0-15.0)
Progression-Free Survival

## Slide 2
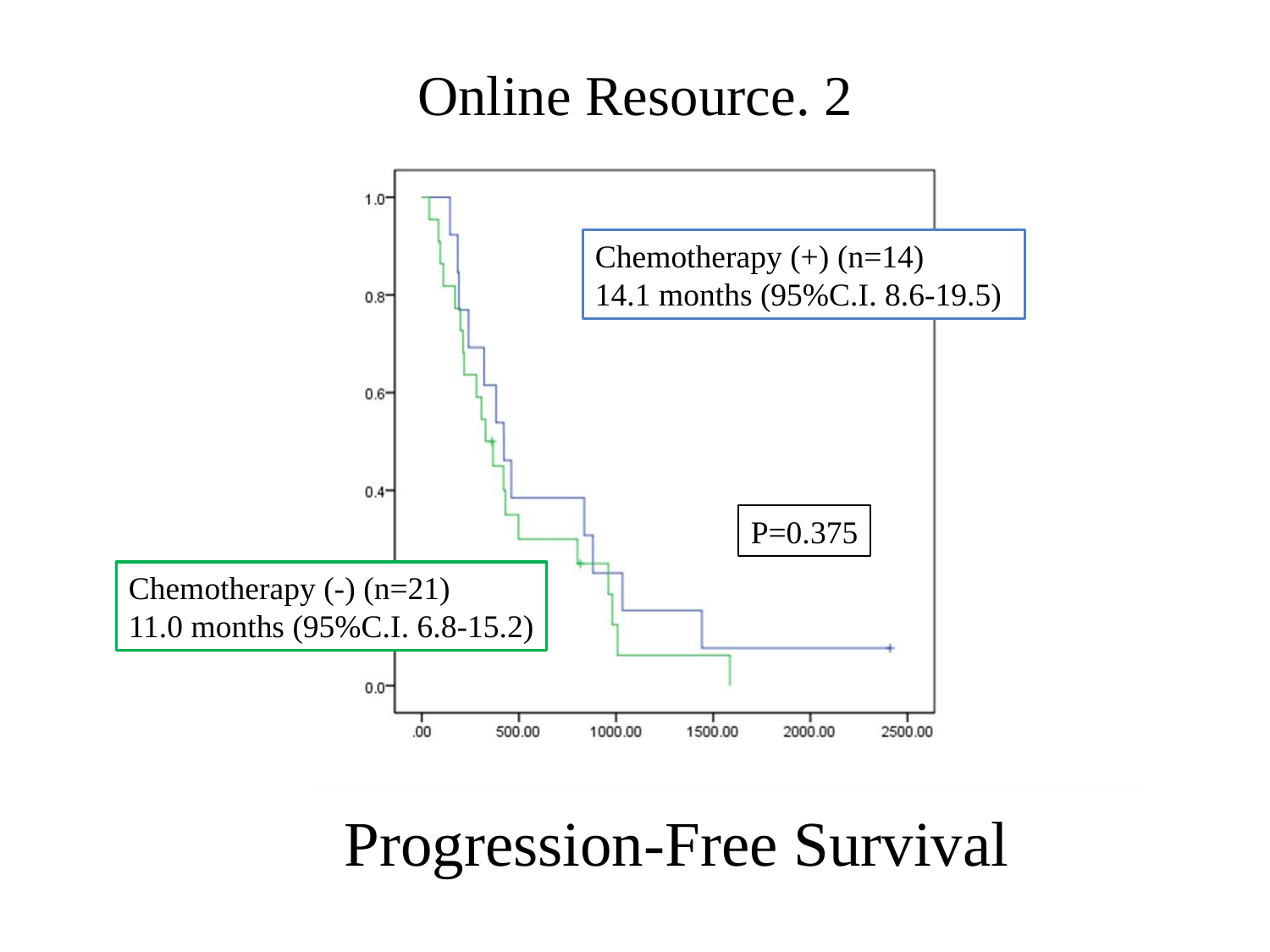

Online Resource. 2
Chemotherapy (+) (n=14)
14.1 months (95%C.I. 8.6-19.5)
P=0.375
Chemotherapy (-) (n=21)
11.0 months (95%C.I. 6.8-15.2)
Progression-Free Survival

## Slide 3
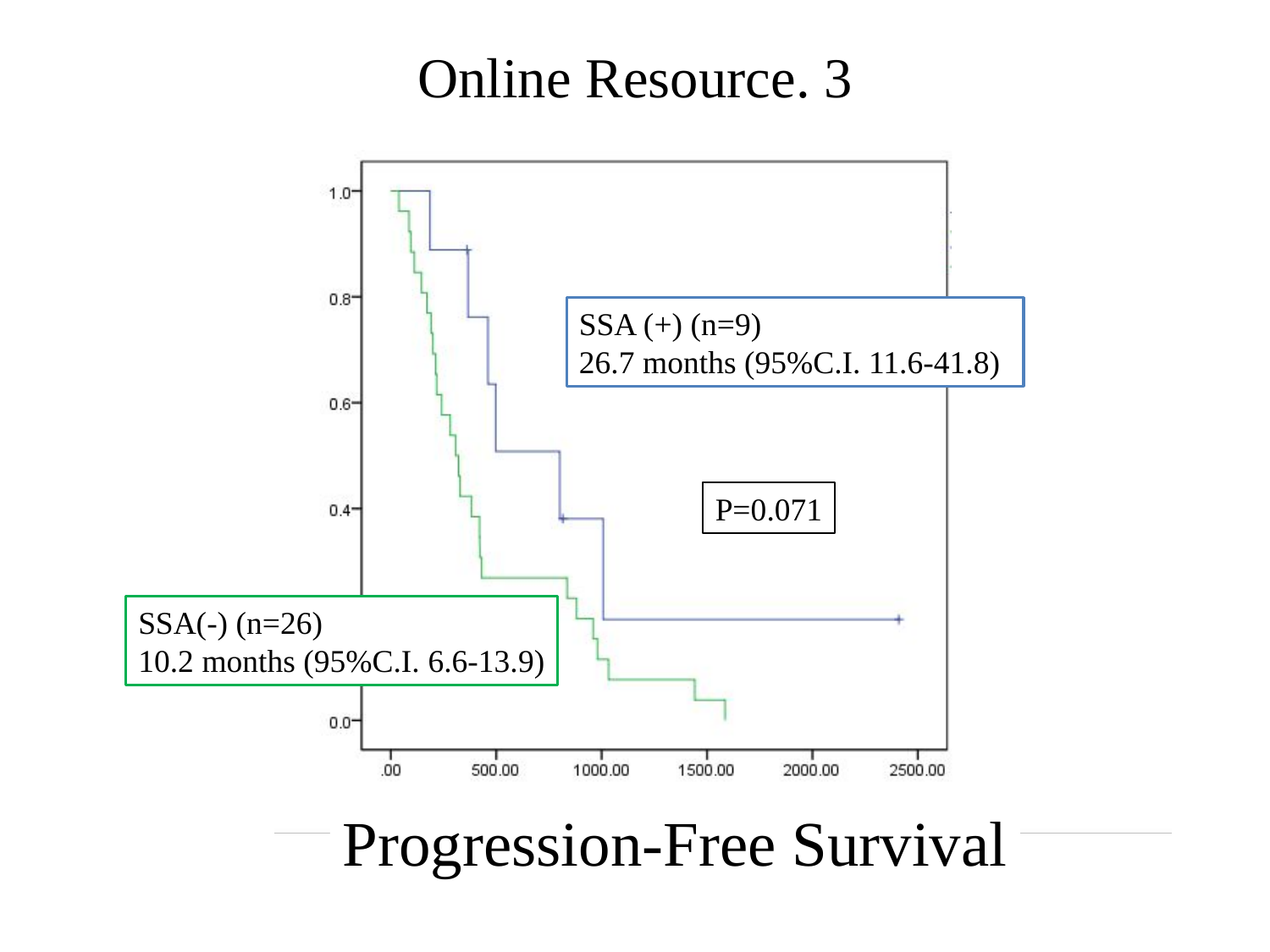

Online Resource. 3
SSA (+) (n=9)
26.7 months (95%C.I. 11.6-41.8)
P=0.071
SSA(-) (n=26)
10.2 months (95%C.I. 6.6-13.9)
Progression-Free Survival

## Slide 4
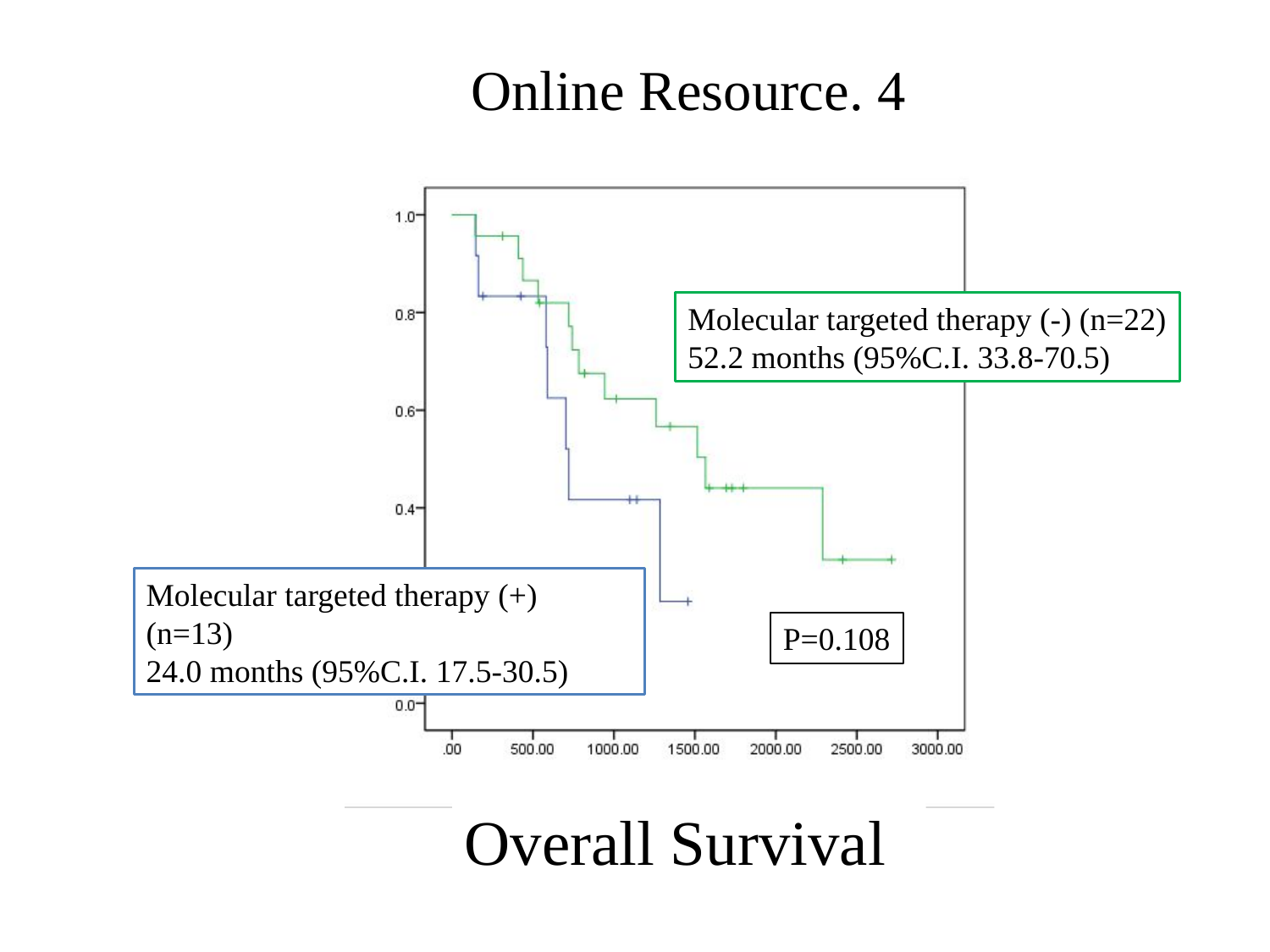

Online Resource. 4
Molecular targeted therapy (-) (n=22)
52.2 months (95%C.I. 33.8-70.5)
Molecular targeted therapy (+) (n=13)
24.0 months (95%C.I. 17.5-30.5)
P=0.108
Overall Survival

## Slide 5
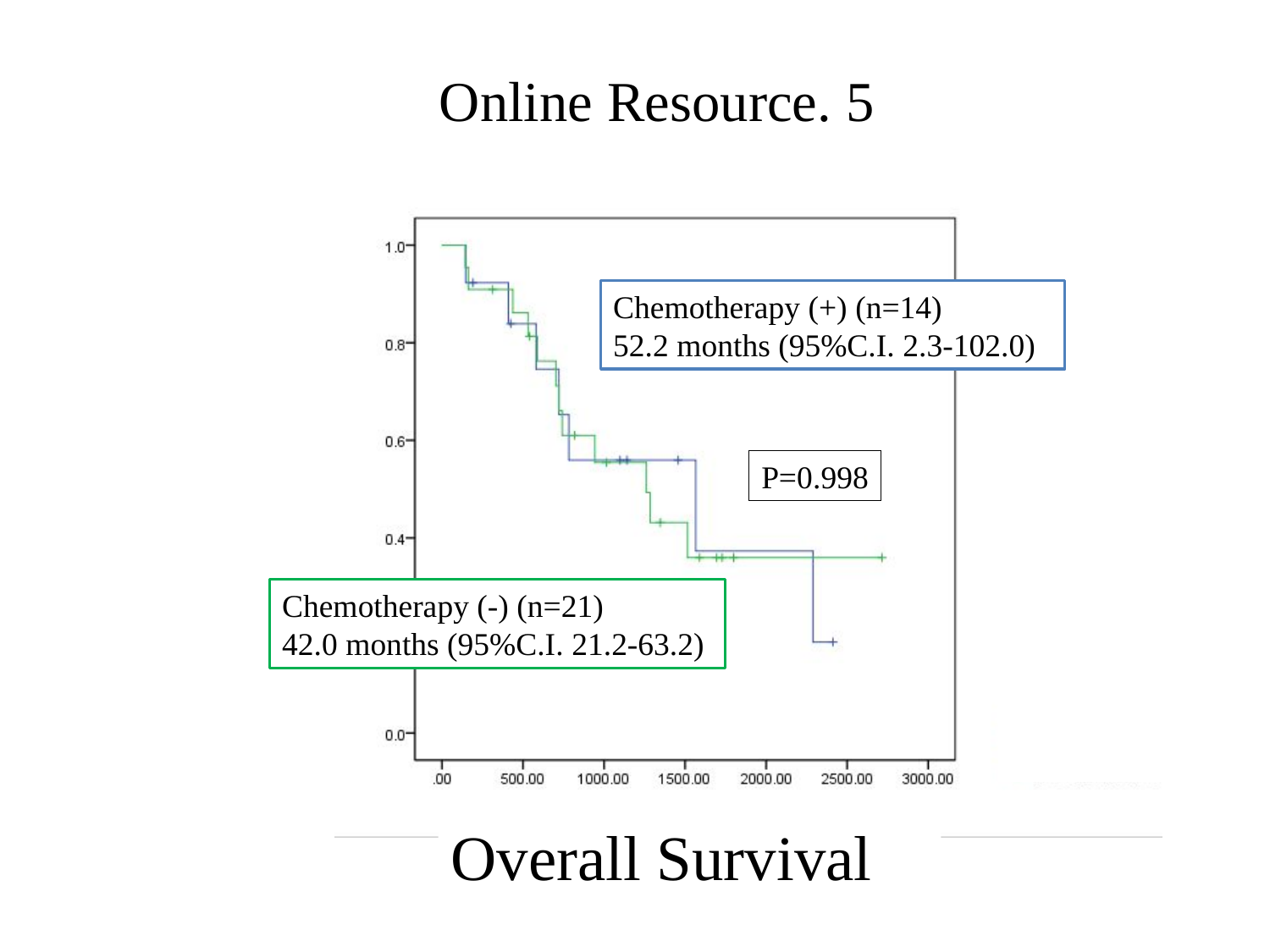

Online Resource. 5
Chemotherapy (+) (n=14)
52.2 months (95%C.I. 2.3-102.0)
P=0.998
Chemotherapy (-) (n=21)
42.0 months (95%C.I. 21.2-63.2)
Overall Survival

## Slide 6
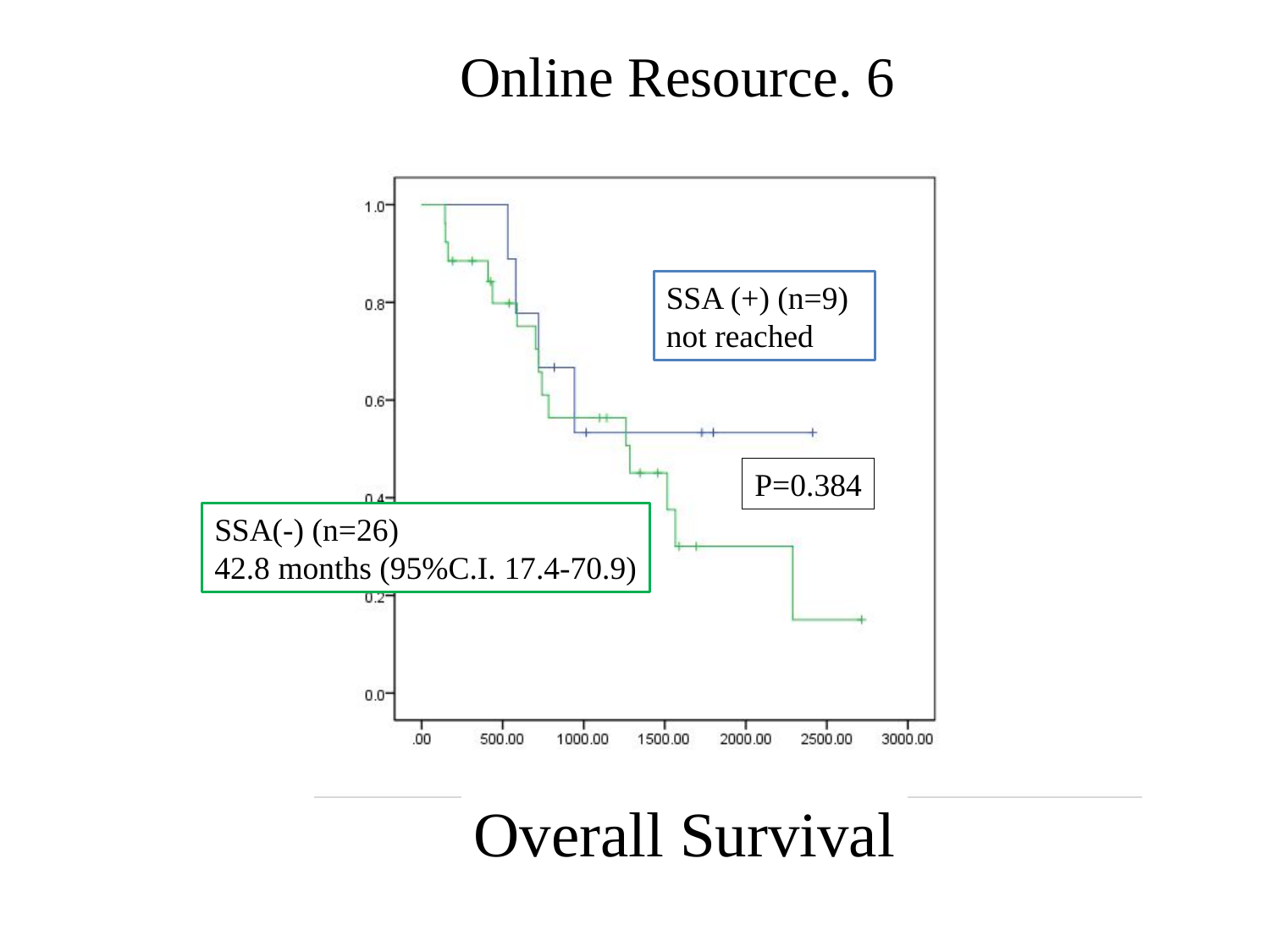

Online Resource. 6
SSA (+) (n=9)
not reached
P=0.384
SSA(-) (n=26)
42.8 months (95%C.I. 17.4-70.9)
Overall Survival
