# Supplementary material for: Retrospective study of peptide receptor radionuclide therapy for Japanese patients with advanced neuroendocrine tumors
Source: J Hepatobiliary Pancreat Sci. 2021 Jul 14;28(9):727–39. doi: 10.1002/jhbp.1014 (PMC9292713; doi:10.1002/jhbp.1014)
Supplement: Supplementary file 2 — Supplementary Material [file JHBP-28-727-s001.docx]

Online Resource 1.

Progression free survival of all patients. Comparison of previous molecular target therapy or not.

Online Resource 2.

Progression free survival of all patients. Comparison of previous chemotherapy or not.

Online Resource 3.

Progression free survival of all patients. Comparison of somatostatin analogs as maintenance treatment or not.

Online Resource 4.

Overall survival of all patients. Comparison of previous molecular target therapy or not.

Online Resource 5.

Overall survival of all patients. Comparison of previous chemotherapy or not.

Online Resource 6.

Overall survival of all patients. Comparison of somatostatin analogs as maintenance treatment or not.
